# Two Novel Rare Strongly Linked Missense SNPs (P27R and A85G) Within the *GDF9* Gene Were Significantly Associated With Litter Size in Shaanbei White Cashmere (SBWC) Goats

**DOI:** 10.3389/fvets.2020.00406

**Published:** 2020-07-30

**Authors:** Yi Bi, Jie Li, Xinyu Wang, Libang He, Kangshu Lan, Lei Qu, Xianyong Lan, Xiaoyue Song, Chuanying Pan

**Affiliations:** ^1^Key Laboratory of Animal Genetics, Breeding and Reproduction of Shaanxi Province, College of Animal Science and Technology, Northwest A&F University, Yangling, China; ^2^College of Veterinary Medicine, Northwest A&F University, Yangling, China; ^3^Shaanxi Provincial Engineering and Technology Research Center of Cashmere Goats, Yulin University, Yulin, China; ^4^Life Science Research Center, Yulin University, Yulin, China

**Keywords:** goat, *growth differentiation factor 9* (*GDF9*) gene, litter size, association, linkage disequilibrium

## Abstract

*Growth differentiation factor 9 (GDF9)* is a high-fertility candidate gene that plays a crucial role in early folliculogenesis in female mammals. In this study, direct sequencing was used to screen possible SNP loci in the goat *GDF9* gene. Three SNP loci, p.proline27alanine (P27R), p.leucine61leucine (L61L), and p.alanine85glycine (A85G), were identified in Shaanbei white cashmere (SBWC) goats. Among the three SNPs, two rare missense SNP loci (P27R and A85G) were discovered to be strongly linked with each other (*D*′ value = 0.926, *r*^2^ value = 0.703). Both P27R and A85G loci had two genotypes: wild type and heterozygous type. A85G exerted a significant effect on litter size (*P* = 0.029) in SBWC goats, and the heterozygous genotype was superior in comparison with the wild type. The heterozygous genotype was also superior in P27R but no significant association was found. However, the combination genotypes of P27R and A85G were identified to have superior effects on litter size (*P* = 3.8E−15). This information suggested that these two SNPs influenced litter size in goats synergistically. Combining this information with our previous studies, we propose that the *GDF9* gene is the principal high-fertility candidate gene and that the A85G locus is a promising SNP that affects litter size in goats. These results may fill a research gap regarding rare mutations as well as provide crucial molecular markers that could be useful in marker-assisted selection (MAS) goat rearing when selecting superior individuals.

## Introduction

The *growth differentiation factor 9* (*GDF9*) gene is a unique member of the transforming growth factor β (TGFβ) superfamily (1), as its protein has six Cys, being different from others in this superfamily that have seven or nine Cys (2, 3). Moreover, its greatest expression is in the ovary while it is widely expressed in 20 different tissues such as the hypothalamus, pituitary, and uterus (4, 5); this indicates that it affects different physiological pathways and metabolism, as well as phenotypic expression to some degree (6). Along with being a powerful intra-ovarian regulator during early folliculogenesis, the *GDF9* gene is expressed throughout follicle development, and its mutations may contribute to increased ovulation rates or infertility in female mammals (7, 8).

Based on relevant study and data from the National Center for Biotechnology Information Search database (NCBI), 45 SNP loci have been identified in the goat *GDF9* gene (9). Among these, 15 SNPs were identified to have significant associations with litter size in more than 30 goat breeds (10–12). Furthermore, there were some controversial and promising SNPs that had different impacts in different goat breeds (13–16). For instance, three missense mutations, A240V (17), Q320P (18–20), and V397I (21, 22), and three synonymous mutations, L61L (23), N121N (24), and L141L (25, 26), were found to have high mutant frequencies and be significantly associated with litter size in different breeds. However, due to breed-specific effects, several of the above results were not consistent. For instance, in Shaanbei white cashmere (SBWC), Lubei White, Jining Gray, Inner Mongolia cashmere, and Laiwu black goat breeds, the G allele is the major allele in V397I (22, 23, 26), but among the Xinong Saanen dairy goat, Big foot black goat, Guanzhong dairy goat, Jintang black goat, and Yimeng black goat, the A allele is associated with larger litter sizes (27, 28). However, almost all the abovementioned studies focused on a single SNP locus and neglected the fact that quantitative traits are controlled by multiple loci. Therefore, the gap in research regarding the combined effects of multiple loci needed to be addressed.

A combination of whole-genome sequencing and marker-assisted selection (MAS) could satisfy the demand to screen pivotal genes accurately and rapidly (29–31), as well as to assess relationships between their variations and growth and reproductive traits (32–35).

As a powerful high-fertility candidate gene, our group previously found that two strongly linked SNPs, Q320P and V397I, and a 12-bp indel within the *GDF9* gene were significant associated with litter size in goats (20, 36). Furthermore, we summarized all reported SNPs within the *GDF9* gene (9). Hence, based on our preliminary work, this study aimed to verify a greater number of SNP loci within the *GDF9* gene as well as to analyze their relationships with goat litter size, thus providing more information for selecting a population with a high fertility using MAS method in goat rearing.

## Materials and Methods

All experiments were approved by the International Animal Care and Use Committee of the Northwest A&F University (IACUC-NWAFU; protocol number NWAFAC1008) and followed local animal welfare guidelines, laws, and policies. The care and use of animals complied with local animal welfare laws and policies.

### Sample Collection and DNA Isolation

For this study, 309 ear tissue samples were randomly collected from female SBWC goats (2–3 years) at a goat-breeding farm in Yulin City, Shaanxi Province, China. All selected goats had the same diet and rearing conditions after weaning (37, 38), were healthy, and had records for their first-born litter size and growth traits (e.g., body height, body length, heart girth, body weight, and cannon bone circumference index). Additionally, random selection ensured that individuals were as unrelated as possible (39, 40).

DNA was extracted from ear tissue samples, diluted to 50 ng/μl, and stored at −20°C according to Aljanabi's method (41). The DNA extraction protocol was as follows: 400 μl of buffer (0.4 M NaCl, 10 mM Tris–HCl at pH 8.0, and 2 mM EDTA at pH 8.0), 40 μl of 20% SDS (2% final concentration), and 8 μl of 20 mg/ml proteinase K (400 μg/ml final concentration) were added to the fresh tissue and mixed well. The samples were kept at a constant temperature of 65°C in a water bath shaker for 12–16 h, followed by the addition of 300 μl of 6 M NaCl (NaCl saturated H_2_O) and subsequent centrifugation for 30 min at 10,000 r/min. The supernatant was then transferred to fresh tubes. An equal volume of isopropanol was added to each sample, mixed well, and samples were incubated at 20°C for 1 h. Samples were then centrifuged for 20 min at 4°C and at 10,000 r/min. The pellet was washed with 70% ethanol, dried, and finally resuspended in 300–500 μl sterile dH_2_O.

### Primer Design, PCR Amplification, and Genotyping

Based on the sequence of *Capra hircus* species (GenBank Accession No. NC_030814.1), a pair of primers (F: 5′-TTTGGTTTTGCTGCTTTGCCT-3′; R: 5′-TCTTTCTTCTTCCCTCCACCCA-3′), which covered P27R, L61L, A85G, L50P, G40G, N112N, and D129D loci, were designed to amplify exon 3 of the goat *GDF9* gene using Primer Premier software (Version 6.0). The PCR was carried out in a 25-μl reaction condition containing 1.0 μl of genomic DNA, 0.5 μl of forward and reverse primer separately, 12.5 μl of 2 × MIX (Tsingke, Xi'an, China), and 10.5 μl of ddH_2_O. The PCR amplification protocol contained a pre-denaturation at 95°C for 5 min and denaturation at 94°C for 30 s, followed by 18 cycles of denaturation for 30 s at 95°C, annealing for 30 s at 68°C (with a decrease of 1°C per cycle), 30 cycles of elongation at 72°C for 30 s, and a final extension at 72°C for 10 min with subsequent cooling to 4°C (42, 43). Subsequently, PCR products were genotyped by electrophoresis using 2.0% agarose gel, which was stained with ethidium bromide. The PCR product was then directly sequenced by the Tsingke Biotechnology Company (Xi'an, China) using Sanger sequencing technology. Finally, sequence alignment was conducted using BioXM 2.6 (College of Agriculture, Nanjing Agricultural University, Nanjing, China) and Chromas 2.4.1 (Technelysium Pty Ltd, South Brisbane, Queensland, Australia).

### Statistical Analyses

Genotype and allele frequencies, Hardy–Weinberg equilibrium (HWE), homozygosity (Ho), heterozygosity (He), effective allele numbers (Ne), and polymorphism information content (PIC) were calculated using PopGene version 1.3.1 (Molecular Biology and Biotechnology Center, University of Alberta, Edmonton, Canada) (44).

Linkage disequilibrium (LD) analysis was conducted on the SHEsis online platform (http://analysis.bio-x.cn) (45). The case of *D*′ = 1 or *r*^2^ = 1 indicated a complete LD. Values of *D*′ < 1; *r*^2^ > 0.33 indicated strong LD (46, 47).

The general linear models were established to analyze correlations between SNP loci and litter size and growth traits using R_3.2.0_ software. For litter size, model I: *Y*_*ijlm*_ = μ + *K*_*i*_ +HYS_*j*_ + *G*_*l*_ + ε_*ijlm*_, where *Y*_*ijlm*_ is the litter size phenotypic value, μ is the mean of the overall population, *K*_*i*_ is the effect of kidding years, HYS_j_ is the mean of population, *G*_*l*_ is the fixed effect of the genotype, and ε_*ijlm*_ is the random error (36).

Considering that growth traits had a positive correlation with litter size (39), association between SNP loci and growth traits were analyzed. The association between SNPs and growth traits was considered. Model II: *Y*_*klm*_ = μ_2_ + *A*_*k*_ + *G*_*l*_ + ε_*klm*_, where *Y*_*klm*_ is the observation of growth traits on each of the *klm*th animal, μ_2_ is the population mean, *A*_*k*_ is the fixed effect of age of the *k*th animal, *G*_*l*_ is the fixed effect of genotypes of the *l*th animal, and ε_*klm*_ is the random error.

*t*-test and the analysis of variance (ANOVA) were conducted to analyze the association between SNP loci and quantitative traits. Moreover, the *t*-test directed to two group analyses and ANOVA were available for >2 group analyses.

### Function Prediction of P27R and A85G Within the Goat *GDF9* Gene

As P27R and A85G are missense SNP loci, they may contribute to amino acid type change during encoding of the goat *GDF9* gene. Herein, potential effects of SNP loci on protein structures and functions were predicted using three prediction tools, SIFT, PolyPhen-2, and PROVEAN (48–50).

## Results

### Genotyping of SNP Loci Within the Goat *GDF9* Gene

**Figure 1:**
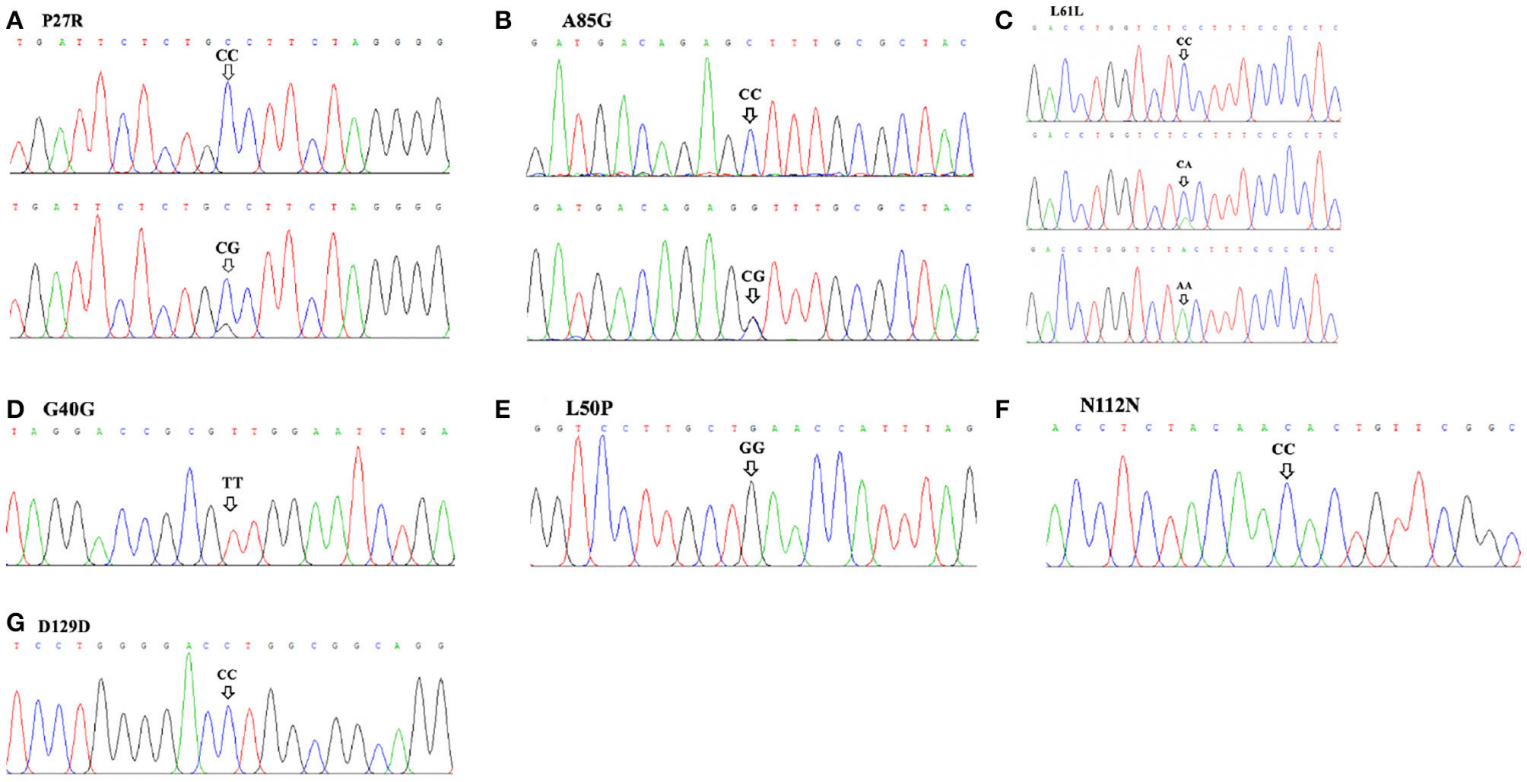
Sequence chromatograms of seven SNPs in the goat *GDF9* gene run on Chromas. **(A)** P27R; **(B)** A85G; **(C)** L61L; **(D)** G40G; **(E)** L50P; **(F)** N112N; **(G)** D129D.

A total of three SNP loci (Table 1; Figure 1) in exon 3 of the *GDF9* gene were detected in this study. Based on sequence chromatograms, three SNP loci (P27R, L61L, and A85G) were genotyped in the analyzed SBWC goat population. For both P27R—-where TGC (proline) transformed to TGG (alanine)—-and L61L loci, two genotypes, CC and CG, were identified. For the A85G locus where CCT (alanine) transformed to CCG (glycine), three genotypes (CC, CA, and AA) were verified.

**Table 1:** Name information of SNPs within the goat *GDF9* gene.

**Names**	**ref SNP No**.	**HGVS names**	**Other names**	**Regions**
SNP1	rs671913497	NC_030814.1: g.66025839C>G	g.1902C>G/c.79C>G/p. P 27R	Exon 3
SNP2	rs669811820	NC_030814.1: g.66025943C>A	g.2006C>A/c.183C>A/p. L61L	Exon 3
SNP3	rs654628150	NC_030814.1: g.66026014C>G	g.2077C>G/c.254C>G/p. A85G	Exon 3

*HGVS, Human Genome Variation Society. The refSNP No. and HGVS names were from the Ensembl database (http://asia.ensembl.org/index.html)*.

### Genotypic and Allelic Frequencies of SNP Loci of the *GDF9* Gene

Based on PIC values, P27R and A85G displayed low genetic diversity and PIC values were 0.047 and 0.019, respectively (Table 2). The PIC value of L61L was 0.355, demonstrating a medium genetic diversity. Additionally, CC and CG genotypes were identified in the P27R locus, and frequencies of C and G alleles were 0.976 and 0.024, respectively. For the L61L locus, three genotypes (CC, CA, and AA) were identified, and frequencies of C and A alleles were 0.639 and 0.361, respectively. For the A85G locus, in which CC and CG genotypes were detected, the frequency of C and G alleles were 0.990 and 0.010, respectively. Furthermore, both P27R and A85G loci met the HWE principle, whereas L61L did not (Table 2).

**Table 2:** Genotypic and allelic frequencies of seven SNPs within the *GDF9* gene in Shaanbei white cashmere (SBWC) goats.

**Loci names**	**Observed genotypes (*N*)**	**Genotype frequencies**	**Allele frequencies**	**Ho**	**He**	**Ne**	**PIC**	**HWE**
P27R	CC (292)	0.951	0.976 (C)	0.952	0.048	1.050	0.047	0.661
	CG (15)	0.049	0.024 (G)					
	GG (0)	0						
L61L	CC (114)	0.373	0.639 (C)	0.539	0.461	1.857	0.355	0.006
	CA (163)	0.533	0.361 (A)					
	AA (29)	0.094						
A85G	CC (297)	0.971	0.990 (C)	0.981	0.019	1.020	0.019	0.727
	CG (12)	0.039	0.010 (G)					
	GG (0)	0						

*Ho, homozygosity; He, heterozygosity; Ne, effective allele numbers; PIC, polymorphism information content; HWE, Hardy–Weinberg equilibrium*.

### Linkage Disequilibrium Analyses

**Figure 2:**
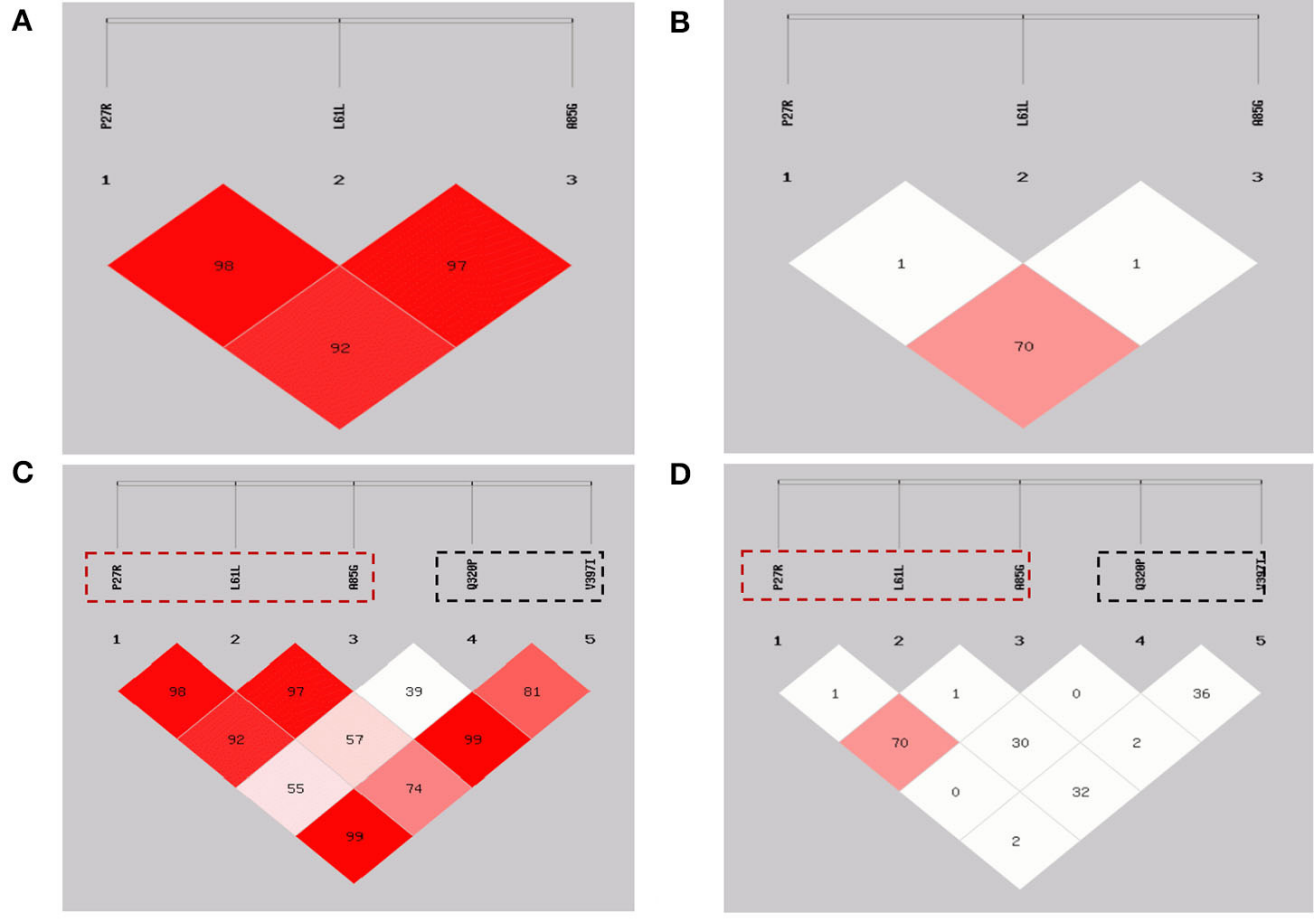
Linkage disequilibrium plots of the *GDF9* gene in Shaanbei white cashmere (SBWC) goats. **(A,C)**: *D*′ of SBWC goats; **(B,D)**: *r*^2^ of SBWC goats. The three loci in the red dotted box were detected in this study and the two loci in the black dotted box were detected in our previous study.

Based on LD analysis results (Table 3; Figure 2), the P27R and A85G loci were discovered to be strongly linked; the *D*′ and *r*^2^ values were 0.926 and 0.703, respectively. For L61L with P27R locus, and L61L with A85G locus, *D*′ values were 0.985 and 0.973, respectively, and *r*^2^ values were 0.016 and 0.012, respectively.

**Table 3:** Linkage disequilibrium parameters (*D*′ and *r*^2^) among P27R, L61L, and A85G loci of the *GDF9* gene in Shaanbei white cashmere (SBWC) goats.

***D*^′^/*r*^2^ value**	**P27R**	**L61L**	**A85G**
P27R	-	0.016	0.703
L61L	0.985	-	0.012
A85G	0.926	0.973	-

*D′ and r^2^ values are shown in the lower and upper triangles of the table, respectively*.

Furthermore, in our previous study, two strongly linked SNPs, Q320P and V397I, were identified to be significantly associated with litter size, using the same population as those in this study (20). Combining our previous data regarding two missense SNPs (Q320P and V397I) within the goat *GDF9* gene (20), the LD analysis of P27R, L61L, A85G, Q320P, and V397I (Table 4; Figure 2) revealed that neither P27R nor A85G was strongly linked to Q320P or V397I; our previous study verified that these were strongly linked, and significantly affected litter size. Interestingly, L61L was almost found to be strongly linked to both the Q320P and V397I loci. For L61L with Q320P, and L61L with V397I, *D*′ values were 0.573 and 0.741, respectively, and *r*^2^ values were 0.306 and 0.324, respectively.

**Table 4:** Linkage disequilibrium parameters (*D*′ and *r*^2^) among P27R, L61L, A85G, Q320P, and V397I loci of the *GDF9* gene in Shaanbei white cashmere (SBWC) goats.

***D*^′^/*r*^2^**	**P27R**	**L61L**	**A85G**	**Q320P**	**V397I**
P27R	-	0.016	0.703	0.005	0.030
L61L	0.985	-	0.012	0.306	0.324
A85G	0.926	0.973	-	0.002	0.025
Q320P	0.557	0.573	0.391	-	0.364
V397I	0.998	0.741	0.998	0.813	-

*D′ and r^2^ values are shown in the lower and upper triangles of the table, respectively*.

### Association Analyses Between SNP Loci and Litter Size

The association analysis between SNP loci and litter size (Table 5) illustrated that the A85G locus was significantly associated with litter size (*P* = 0.029), with the heterozygosity genotype displaying a superior phenotype over the homozygosity genotype. However, the P27R locus did not exert a remarkable effect on litter size. In combination genotype analysis of the P27R and A85G loci (Table 6), combination genotypes were significantly correlated to litter size (*P* = 3.8E−15) and the CG-CG produced the largest litter size. Furthermore, based on our previous data of Q320P and V397I (20), we did a combination genotype analysis, which showed that combination genotypes of P27R, A85G, Q320P, and V397I also exerted significant impacts on litter size (*P* = 0.001) with the CC-CC-CC-AA being dominant.

**Table 5:** Relationship between SNP loci of the *GDF9* gene and litter size in Shaanbei white cashmere (SBWC) goats.

**SNP loci**	**Genotypes**	**Litter size (*n*)**	***P*-values**
P27R	CC	^a^1.70 ± 0.48 (*n* = 292)	0.404
	CG	^a^1.80 ± 0.41 (*n* = 15)	
A85G	CC	^b^1.70 ± 0.48 (*n* = 297)	**0.029**
	CG	^a^1.91 ± 0.28 (*n* = 12)	

*Values with different letters (a,b) within the same row differ significantly at P < 0.05*.

**Table 6:** Least squares mean and standard error for litter size of different combination genotypes of the *GDF9* gene in Shaanbei white cashmere (SBWC) goats.

**SNP loci**	**Genotypes**	**Litter size**	***P*-values**
Combination genotypes with the P27R and A85G	CC-CC	^B^1.72 ± 0.02 (*n* = 300)	**0.036**
	CG-CG	^A^1.77 ± 0.22 (*n* = 13)	
Combination genotypes with the P27R, A85G, Q320P, and V397I	CC-CC-AA-AG	^A^1.77± 0.07 (*n* = 30)	**0.001**
	CC-CC-AA-GG	^A^1.69± 0.07 (*n* = 42)	
	CC-CC-AC-AA	^B^1.52± 0.10 (*n* = 23)	
	CC-CC-AC-AG	^A^1.75± 0.05 (*n* = 64)	
	CC-CC-CC-AA	^A^1.78± 0.10 (*n* = 18)	

*Values with different letters (A, B) within the same row differ significantly at P < 0.01*.

### Association Analyses Between SNP Loci and Growth Traits

Previous studies by our group report a significant positive correlation between litter size and growth traits in our SBWC goat cohorts (20, 36); therefore, the relationship between these two missense SNPs (P27R and A85G) was addressed here. Association analyses between P27R and A85G loci as well as growth traits of SBWC goats revealed that the A85G locus was significantly related to body length (*P* = 0.001) and heart girth (*P* = 0.002). Furthermore, the heterozygosity genotype was superior when compared with the wild type (Table 7). No significance was found for P27R and growth traits. However, combination genotypes of P27R and A85G were proved to be significantly associated with hip width (*P* = 6.9E−8) and the cannon circumference index (*P* = 1.0E−6), with the phenotype of the homozygosity genotype showing a superior performance over the heterozygosity genotype (*P* = 6.9E−8; Table 8).

**Table 7:** Relationship between the A85G locus of the *GDF9* gene and growth parameters in Shaanbei white cashmere (SBWC) goats.

**Parameters**	**Genotypes (*N*)**		***P*-values**
	**CC**	**CG**	
BL (cm)	^B^68.72 ± 0.26 (*n* = 288)	^A^71.33 ± 0.58 (*n* = 12)	0.001
HG (cm)	^B^90.14 ± 0.47 (*n* = 297)	^A^95.17 ± 1.28 (*n* = 12)	0.002

*BL, body length; HG, heart girth. Values with different letters (A, B) within the same row differ significantly at P < 0.01*.

**Table 8:** Least squares mean and standard error for growth parameters of different combination genotypes of the SNPs P27R and A85G within the *GDF9* gene in Shaanbei white cashmere (SBWC) goats.

**Parameters**	**Genotypes (*N*)**		***P*-values**
	**CC-CC**	**CG-CG**	
HW (cm)	^A^31.67 ± 1.85 (*n* = 283)	^B^20.15 ± 0.61 (*n* = 13)	6.9E−8
CCI (%)	^A^19.77 ± 0.76 (*n* = 275)	^B^14.89 ± 0.45 (*n* = 13)	1.0E−6

*HW, hip width; CCI, cannon circumference index. Values with different letters (A, B) within the same row differ significantly at P < 0.01*.

### Protein Function Prediction of SNP Loci

For protein function prediction of the P27R and A85G loci, the scores of SIFT, PolyPhen-2, and PROVEAN suggested that their influence on the GDF9 protein was benign and probably damaging, respectively. The scores implied that A85G had an impact on protein structure change of the goat *GDF9* gene.

## Discussion

The *GDF9* gene plays a considerable role in the control of somatic cell functions such as follicular proliferation, ovulation, and fertilization, as well as enhancing oocyte development in females (51, 52). Therefore, study of the *GDF9* gene and its mutations is worthwhile (53, 54). In the current study, three SNP loci (P27R, L61L, and A85G) were identified within the goat *GDF9* gene. The L61L locus is reported to have a negative association with litter size in Jining Gray and Yimeng Black goat breeds (55). However, no significant association has been noted in Wendeng dairy, Liaoning cashmere, Beijing native, Boer, and Lubei goat breeds (23), which was consistent with the findings of this study. This may be due to a breed-specific effect or some degree of linkage between L61L and other SNP loci (22). Furthermore, two novel SNP loci, P27R and A85G, were detected to have low mutation frequencies and be strongly linked. The A85G locus was found to be significantly associated with litter size in SBWC goats while the P27R locus was not. However, combination genotypes of P27R and A85G showed significant association with litter size of SBWC goats with the heterozygosity genotype having a larger litter size. This could imply that these two SNP loci may exert a synergistic effect on litter size in goats. Considering that the SIFT, PolyOhen-2, and PROVEAN scores of P27R and A85G were benign and probably damaging, respectively, A85G might be responsible for changes in protein structure as well as DNA-binding ability. Additionally, this variation may alter *GDF9* gene outcomes in terms of post-transcriptional regulation, such as RNA modification (56, 57), alternative splicing (58), and tRNA processing (56). For P27R, which is suggested to have no effect on changes in protein structure, it might be linked with other major polymorphisms exerting indirect effects (20).

In our previous studies, a 12-base pair indel (36) and two strongly linked SNP loci, Q320P and V397I, were significantly associated with litter size in goats (20). Q320P&V397I and P27R&A85G were regarded as block1 and block2, respectively. To investigate the relationship between block1 and block2, LD analyses were performed. Results illustrated that no strong linkage existed between the two blocks, but the combination genotype of the Q320P&V397I&P27R&A85G loci was significantly associated with litter size in goats, and it could be speculated that the two blocks affected litter size synchronously. Given the fact that quantitative traits are controlled by multiple loci, and only two strongly linked blocks have been uncovered to date (20), the identification of novel strongly linked loci, as well as their combined effects, would be promising for MAS breeding.

Already knowing that growth traits are positively correlated with reproductive traits (39), our study set out to determine whether the two strongly linked SNPs had a significant effect on growth traits. Association analyses showed that A85G was significantly associated with growth traits, but this was not true of P27R. However, combination genotype analyses further verified that combination genotypes of P27R and A85G exerted a significant effect on growth traits. These findings hinted that P27R and A85G might have a synergistic effect on growth performance. Therefore, both A85G and P27R could be considered as growth-related loci to some degree.

## Conclusion

In conclusion, two novel rare missense SNPs were verified to be strongly linked in this study. Moreover, A85G was significantly associated with litter size, and P27R could have a simultaneous effect. The present study succeeded in establishing a clear and significant correlation between two SNPs (P27R and A85G) within the *GDF9* gene with litter size in SBWC goat. Further studies are needed to identify sire effect before the commercial use of these SNPs in MAS.

## Data Availability Statement

The datasets analyzed for this study can be found in the Ensembl database under accession numbers listed in Table 1 [http://www.ensembl.org/index.html].

## Ethics Statement

The animal study was reviewed and approved by The International Animal Care and Use Committee of the Northwest A&F University (IACUC- NWAFU) (protocol number NWAFAC1008). Written informed consent was obtained from the owners for the participation of their animals in this study.

## Author Contributions

YB, JL, and XW came with idea and wrote manuscript. LH, KL, XS, and XL collected the goat samples and isolated of genomic DNA. YB, JL, XW, and LH performed the experiments. YB, LQ, XS, XL, and CP analyzed the data. All authors approved the final version of the manuscript for submission. All authors contributed to the article and approved the submitted version.

## Conflict of Interest

The authors declare that the research was conducted in the absence of any commercial or financial relationships that could be construed as a potential conflict of interest.

## Abbreviations

GDF9: growth differentiation factor 9
SNPs: single-nucleotide polymorphisms
PCR: polymerase chain reaction
SBWC: Shaanbei white cashmere goat
P27R: p.proline27alanine
L61L: p.leucine61leucine
A85G: p.alanine85glycine
Q320P: p.glutamine320proline
V397I: p.valine397isoleucine
A240V: p.alanine240valine
N121N: p.asparagine336asparagine
L141L: p.leucine141leucine
LD: linkage disequilibrium
HWE: Hardy–Weinberg equilibrium
Ho: homozygosity
He: heterozygosity
Ne: effective allele number
PIC: polymorphism information content
MAS: marker-assisted selection.
